# Dual-modality imaging with ^99m^Tc and fluorescent indocyanine green using surface-modified silica nanoparticles for biopsy of the sentinel lymph node: an animal study

**DOI:** 10.1186/2191-219X-3-33

**Published:** 2013-04-25

**Authors:** Makoto Tsuchimochi, Kazuhide Hayama, Michio Toyama, Ichiro Sasagawa, Norio Tsubokawa

**Affiliations:** 1 Department of Oral and Maxillofacial Radiology, The Nippon Dental University School of Life Dentistry at Niigata, 1-8 Hamaura-cho, Chuo-ku, Niigata, Niigata, 951-8580, Japan; 2 Advanced Research Center, The Nippon Dental University School of Life Dentistry at Niigata, Niigata, Niigata, 951-8580, Japan; 3 Faculty of Engineering, Niigata University, 8050 Ikarashi 2-no-cho, Nishi-ku, Niigata, Niigata, 950-2181, Japan

**Keywords:** Multimodality imaging, Sentinel lymph node, ^99m^Tc, Near-infrared fluorescence, ICG

## Abstract

**Background:**

We propose a new approach to facilitate sentinel node biopsy examination by multimodality imaging in which radioactive and near-infrared (NIR) fluorescent nanoparticles depict deeply situated sentinel nodes and fluorescent nodes with anatomical resolution in the surgical field. For this purpose, we developed polyamidoamine (PAMAM)-coated silica nanoparticles loaded with technetium-99m (^99m^Tc) and indocyanine green (ICG).

**Methods:**

We conducted animal studies to test the feasibility and utility of this dual-modality imaging probe. The mean diameter of the PAMAM-coated silica nanoparticles was 30 to 50 nm, as evaluated from the images of transmission electron microscopy and scanning electron microscopy. The combined labeling with ^99m^Tc and ICG was verified by thin-layer chromatography before each experiment. A volume of 0.1 ml of the nanoparticle solution (7.4 MBq, except for one rat that was injected with 3.7 MBq, and 1 μg of an ICG derivative [ICG-sulfo-OSu]) was injected submucosally into the tongue of six male Wistar rats.

**Results:**

Scintigraphic images showed increased accumulation of ^99m^Tc in the neck of four of the six rats. Nineteen lymph nodes were identified in the dissected neck of the six rats, and a contact radiographic study showed three nodes with a marked increase in uptake and three nodes with a weak uptake. NIR fluorescence imaging provided real-time clear fluorescent images of the lymph nodes in the neck with anatomical resolution. Six lymph nodes showed weak (+) to strong (+++) fluorescence, whereas other lymph nodes showed no fluorescence. Nodes showing increased radioactivity coincided with the fluorescent nodes. The radioactivity of 15 excised lymph nodes from the four rats was assayed using a gamma well counter. Comparisons of the levels of radioactivity revealed a large difference between the high-fluorescence-intensity group (four lymph nodes; mean, 0.109% ± 0.067%) and the low- or no-fluorescence-intensity group (11 lymph nodes; mean, 0.001% ± 0.000%, *p* < 0.05). Transmission electron microscopy revealed that small black granules were localized to and dispersed within the cytoplasm of macrophages in the lymph nodes.

**Conclusion:**

Although further studies are needed to determine the appropriate dose of the dual-imaging nanoparticle probe for effective sensitivity and safety, the results of this animal study revealed a novel method for improved node detection by a dual-modality approach for sentinel lymph node biopsy.

## Background

Sentinel lymph node biopsy has been widely used in cancer treatment to assess lymph node metastasis at early stages. It has been reported that this procedure has an accuracy rate greater than 95% in the evaluation of lymph node metastasis in patients with stage N0 carcinoma, especially in patients with malignant melanoma or breast carcinoma. Sentinel lymph nodes are those that directly drain a primary tumor, and metastatic cells are likely to spread to and lodge in the sentinel lymph node(s) first. Therefore, precise examination of the sentinel lymph node can accurately predict the lymph node status of the entire lymph basin at early stages of cancer progression.

Before a biopsy is taken, lymphoscintigraphy (sentinel lymph node mapping) is performed to identify the pattern of the lymphatic drainage and the sentinel lymph nodes associated with the primary tumor. Lymphoscintigraphy can reveal unexpected lymphatic vessels and lymph nodes associated with different lymphatic drainage basins. Surgeons trace the sentinel lymph node using a gamma probe that can provide radioactivity counts. Variable-pitch audio output is based on the intensity of the radioactivity and is detected in the operating theater. As surgeons are unable to visualize increased radioactivity with spatial resolution, a small gamma camera can be used for this purpose at the time of the biopsy [1]. However, sentinel lymph nodes with increased accumulation cannot be defined with anatomical resolution. The blue-dye technique is often combined with the radioisotope method to yield greater sentinel lymph node detection [2]. However, the blue-dye technique only offers the visualization of lymph flow and sentinel nodes within the incision area. Furthermore, these blue dyes are soluble and are quickly flushed from lymphatic channels. The lymphatic flow containing the dye may disappear rapidly from the lymph nodes. When performed alone, this method has a much lower sentinel lymph node identification rate compared to lymphoscintigraphy [2].

Attempts to combine several effective techniques have been reported in animal studies, including the addition of technetium-99m (^99m^Tc), ^125^I, or ^111^In to approaches using Evans Blue [3], methylene blue [4], phthalocyanine tetrasulfonate [5], dextran, blue Ficoll dyes, among others. In addition, a study by Tsopelas et al. [3] reported that ^99m^Tc-Evans Blue was useful for differentiating the initial draining lymph node from higher-tier nodes in linked chains. This study also demonstrated the advantage of providing radioactive and color signals simultaneously during operative exposure.

Near-infrared (NIR) imaging using indocyanine green (ICG) was recently introduced as a new option for imaging sentinel lymph nodes [6-8]. ICG has a long history of use in the clinic for testing liver function and cardiac output and for ophthalmic angiography. Recent studies have demonstrated the usefulness of ICG fluorescence imaging during sentinel lymph node biopsy in comparison to other visual dyes. This efficacy probably stems from the advantages of NIR fluorescence, which allows deeper penetration of photons into living tissue due to its decreased absorption and scattering and minimal tissue autofluorescence at the wavelengths utilized [9]. However, tissue penetration is still limited compared to gamma rays.

Recently, the combination of NIR and radionuclide imaging was tested to reveal sentinel lymph nodes in animals. In this method, albumin radiocolloid (^99m^Tc) and ICG are conjugated for lymphoscintigraphy and fluorescent imaging simultaneously [10]. This new combined imaging has also been introduced into clinical settings [11,12]. A successful multimodal sentinel lymph node imaging with NIR agent (Cy7) covalently labeled with ^99m^Tc has also been reported in animals [13].

We sought to develop a new approach by silica nano-particle-based dual-modality imaging to facilitate sentinel node biopsy examinations, in which radioactive and NIR fluorescent nanoparticles are used to depict deeply situated sentinel nodes and fluorescent nodes and to enable simultaneous visualization at anatomical resolution within the field of interest. For this purpose, we developed polyamidoamine (PAMAM)-coated silica nanoparticles loaded with ^99m^Tc and ICG and conducted animal studies to test the feasibility and utility of this dual-modality imaging system.

## Methods

We developed PAMAM-coated silica nanoparticles (PCSNs) loaded with ^99m^Tc radioactivity and NIR fluorescent ICG for dual-modality imaging to detect sentinel lymph nodes in basic and animal studies.

### PAMAM-coated silica nanoparticles

**Figure 1:**
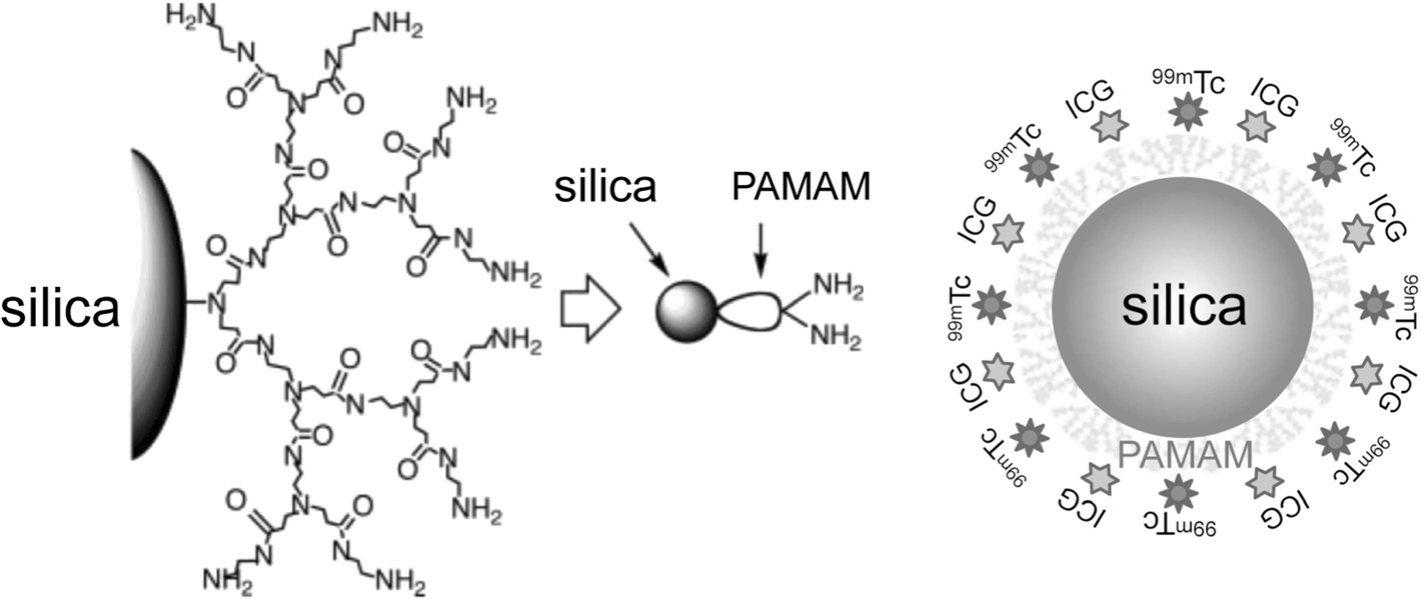
**PAMAM-coated silica nanoparticles loaded with **^**99m**^**Tc and ICG.**

These studies were performed using Aerosil 200 synthetic amorphous silica nanoparticles obtained from Nippon Aerosil, Tokyo, Japan. The particle size, specific surface area, and silanol group content were 12 nm, 200 m^2^/g, and 1.37 mmol/g, respectively [14]. The grafting of hyperbranched PAMAM (generation 3) onto the silica nanoparticle surface was achieved, as shown in Figure 1[15].

The percentage of grafted PAMAM and the amino group content of the silica nanoparticles used in these experiments were 55.6% and 3.6 mmol/g, respectively. The percentage of PAMAM that grafted onto the silica surface was determined using a previously described equation [14]. The amino group content on the silica nanoparticle surface was determined by titration.

### Loading of ^99m^Tc and ICG

Twenty milligrams of PCSNs was diluted in 50% ethanol with distilled water (total volume, 20 ml) and mixed using an ultrasonic water bath for 30 min. The diluted solution was filtered using a centrifugal device (ultrafree centrifugal filter, Durapore PVDF 0.1 μm, Millipore, Billerica, MA, USA) and a centrifuge with a radius of 15.0 cm (13,000 rpm, 15 min). After separating the smaller particles (less than 0.1 μm), the filtrates were used for further processing.

^99m^Tc (74 MBq/100 μl) was added to 890 μl of the filtrate for chelation to the surface of the nanoparticles; this solution was then incubated for 30 min at 37°C.

One milligram of ICG-sulfo-OSu (2-[7-[1,3-dihydro-1,1-dimethyl-3-(4-sulfobutyl)-2*H*-benzo[e]indol-2-ylidene]-1,3,5-heptatrienyl]-1,1-dimethyl-3-[5-(3-sulfosuccinimidyl)oxycarbonylpentyl]-1*H*-benzo[e] indolium, inner salt, and sodium salt; Dojindo Laboratories, Kumamoto, Japan) was dissolved in 1 ml of DMSO (Thermo Scientific, Hudson, NH, USA). Ten microliters of the ICG-sulfo-OSu dilution was added to 990 μl of the solution containing the radiolabeled nanoparticles for N-terminal binding to the surface of the PCSNs. The solution of nanoparticles, which was now loaded with radioactivity and fluorescence, was used as a probe in the subsequent experiments (Figure 1). The PCSN probe solutions were analyzed by silica concentration measurement. The samples were evaporated to dryness with an alkali fusion procedure. This was followed by inductively coupled plasma-atomic emission spectrometry (ICP-AES) (iCAP 6500 Duo, Thermo Fisher Scientific Inc., Waltham, MA, USA) measurement. The zeta potential of the prepared PCSN was measured using an automated electrokinetic analyzer (ELSZ-2N, Otsuka Electronics Co. Ltd., Osaka, Japan). The measurement was repeated five times.

### Size of PCSNs

The size of the PCSNs prepared was evaluated using a transmission electron microscope (TEM) and a scanning electron microscope (SEM; S-800 15kV, Hitachi, Tokyo, Japan). The size distribution and concentration of the nanoparticles in the solution were assessed using an optical particle-size analyzer (SALD-7100, Shimadzu, Kyoto, Japan).

### Radioactivity and fluorescence of the PCSN probe

The radioactivity and fluorescence of the labeled PCSNs were verified with thin-layer chromatography (TLC) utilizing TLC silica gel 60 F254 (Merck KGaA, Darmstadt, Germany) with an imaging plate (BAS-SR2040, FujiFilm, Tokyo, Japan) and using a fluorescence imaging system (photodynamic eye (PDE); Hamamatsu Photonics, Hamamatsu, Japan) or ImageQuant LAS-4000 (GE Healthcare, Piscataway, NJ, USA). TLC was performed using 2 μl of the PCSN probe solution applied to the TLC plate, and the radioactivity and fluorescence were quantified 25 min later. TLC contact radiography was performed by placing the TLC plates on the imaging plate and then scanning them using a phosphor imager FLA-2000 (FujiFilm, Tokyo, Japan). Fluorescence levels were visualized from the display.

### Animal experiments

Six male Wistar rats (weight: mean, 530 g; range, 480 to 560 g; Charles River Breeding Laboratories, Yokohama, Japan) were anesthetized via an intraperitoneal injection of sodium pentobarbital (30 to 40 mg/kg) (Somnopentil, Schering-Plough Animal Health, Omaha, NE, USA). After the rats had been immobilized on a table, 0.1 ml of the PCSN probe solution (7.4 MBq, except for one rat that was injected with 3.7 MBq, and 1 μg ICG) was injected submucosally into the tongue. Subsequent scintigraphic studies used a gamma camera (SNC-5100R, matrix size of 512 × 512 pixels, field of view 51 × 38 cm, parallel-hole low-energy (high resolution) collimator; Shimadzu, Kyoto, Japan). One of the six rats was injected in the forefoot with the PCSN probe solution; these results were not analyzed.

Lymphoscintigraphy (sequential static images, every 10 min) was performed in all six rats during the first hour after injection. After completion of the lymphoscintigraphy, the animals were euthanized via intraperitoneal injection of an overdose of sodium pentobarbital. Immediately after euthanasia, a skin flap was created to expose the underlying tissue of the neck. The sentinel nodes were explored with NIR fluorescence imaging using a PDE fluorescence imaging system, which included a digital camera with a charge-coupled device detector that filtered out light wavelengths <820 nm. The resulting fluorescent images were displayed on a monitor. All lymph nodes with or without fluorescence were dissected for subsequent examination. In some rats, a small cadmium telluride (CdTe) gamma camera (SSGC) was used for additional imaging of the sentinel lymph nodes before dissection. The head of the SSGC consisted of a ‘pixelized’ CdTe module (32 × 32 individual elements, total of 1,024 pixels) attached to a collimator (Acrorad, Tokyo, Japan). The field of view was 44.8 mm × 44.8 mm [16,17]. The excised lymph nodes were examined using a PDE imaging system or the LAS 4000 for fluorescence imaging. The intensity of the fluorescence was qualitatively scored as no fluorescence (−), weak (+), medium (++), or strong (+++).

Autoradiography was performed using FLA-2000 with an imaging plate (IP). The excised lymph nodes were weighed and placed on the IP, which was covered with a sheet of polyvinylidene film. The samples were exposed on the IP in a darkroom for 5 min to produce autoradiographic images of the radioactivity, and the IP was then processed to image the radioactivity using the FLA-2000 device.

Radioactivity in the lymph nodes and in a set of reference samples of ^99m^Tc was assayed using an autowell gamma system (ARC-370 M, Aloka, Tokyo, Japan). Radioactivity was corrected for decay time, and it is expressed as percentages of the activity in the specimens relative to the initially injected radioactivity. Percentage variability data are expressed as mean ± SD.

All animals were treated in accordance with the Ethical Guidelines for Investigations of Experimental Animals of the Nippon Dental University School of Life Dentistry at Niigata.

### Transmission electron microscopy

Excised specimens were confirmed as lymphatic nodes using H&E staining and toluidine blue staining. Seven lymph nodes were examined using a TEM (H-7000, Hitachi, JEM1010; JEOL, Tokyo, Japan) operated at 80 kV and a JEM2010 TEM (200 kV) equipped with an energy-dispersive spectroscopy (EDS) system (JEOL, Tokyo, Japan). Resin blocks were made from formalin-fixed tissue, and semithin and ultrathin sections were then cut for observation.

### Statistics

Two-group comparisons were performed using Welch's *t*-test. SPSS software v. 15.0J (IBM SPSS, Tokyo, Japan) was used for these analyses.

## Results

**Figure 2:**
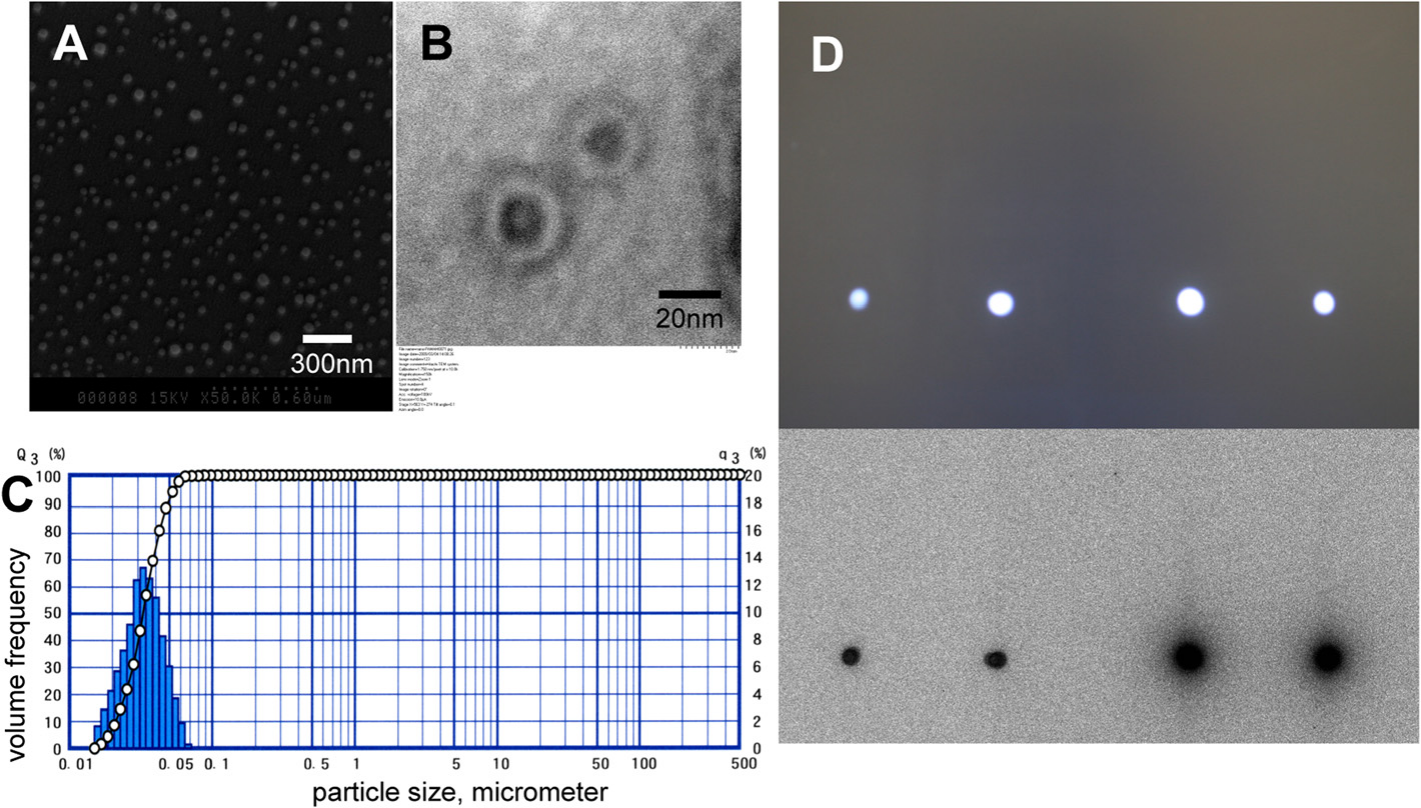
**SEM and TEM images of silica nanoparticles, distribution of particle sizes, and combined labeling.** The mean diameter of the PAMAM-coated silica nanoparticles was 20 to 50 nm, as evaluated by scanning electron microscopy (**A**) and transmission electron microscopy (**B**). The distribution of the particle sizes was measured using a laser-scattering particle-size analyzer (**C**). The peak of the distribution was 30 nm in ethanol solution. Combined labeling with both ^99m^Tc and ICG-sulfo-OSu for PCSNs was verified by TLC before each experiment (**D**). Two-microliter spots of the grafted PCSNs were visualized on TLC plates for NIR fluorescence imaging (above) and contact radiography (below). Verification of the complete graft was confirmed by the lack of flares on either plate.

The mean diameter of the PCSNs was 30 to 50 nm as evaluated by TEM and SEM (Figure 2A,B). The distribution of the particle sizes was measured using a laser-scattering particle size analyzer, SALD-7100. The peak of the distribution was 30 nm in the ethanol solution (50% water; Figure 2C). Combined labeling of the PCSN probes with both ^99m^Tc and ICG-sulfo-OSu was verified by TLC before each experiment. Areas containing 2 μl of the grafted PCSN probes were visualized for radioactivity by contact radiography and for fluorescence using the LAS-4000 in exactly the same location. Flares caused by free agents were not observed in either of the confirmation procedures (Figure 2D). The concentration of silica in the PCSN probe solution was 3 mcg/g (ppm) by ICP-AES. The mean of the zeta potential of PCSN was determined to be 8.09 ± 0.24mV (mean ± SD) using the Smoluchowski equation. Nanoparticles with zeta potentials greater than +30 mV are considered as strongly cationic. The PCSN is considered approximately neutral because the zeta potential is in a range extending between −10 and +10 mV.

**Figure 3:**
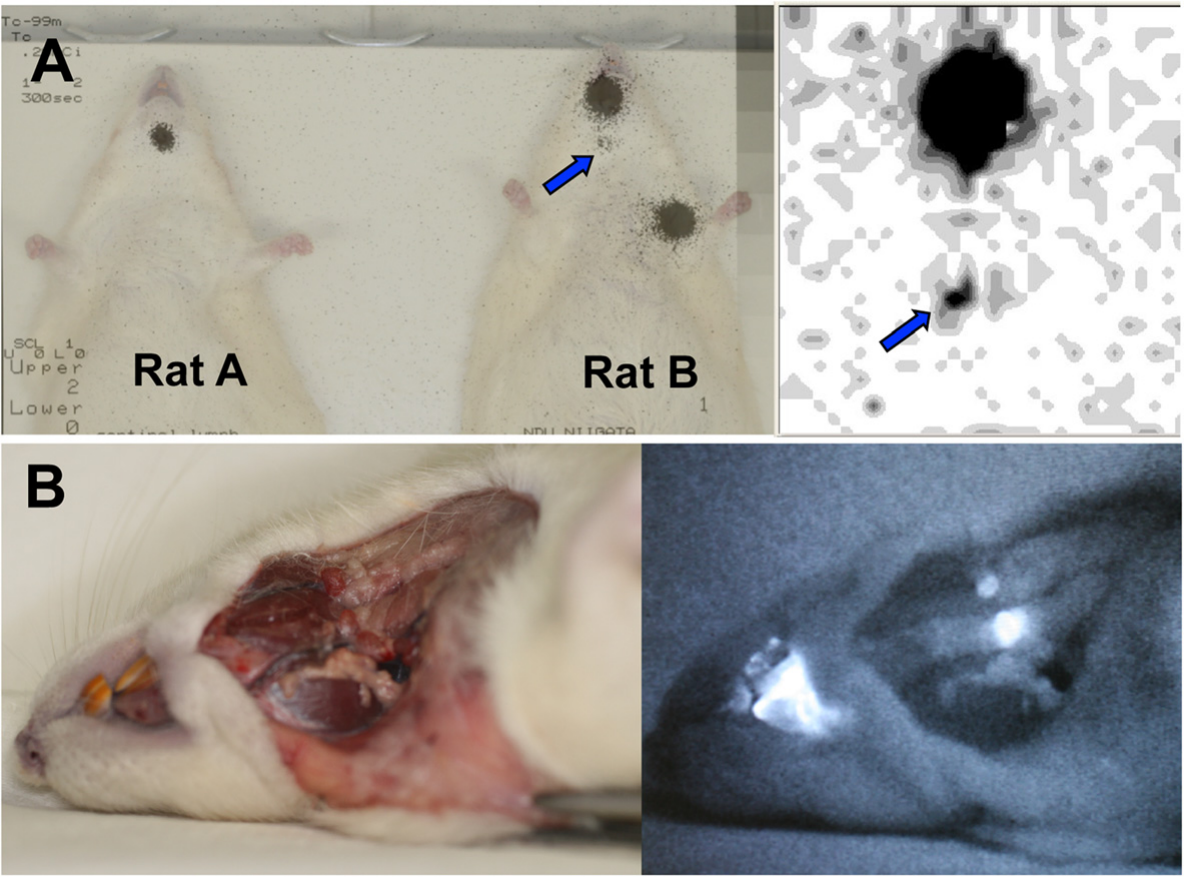
**Lymphoscintigraphy of the rats and lymph node dissection in the neck.** Lymphoscintigraphy of the rats (**A**, left). The nanoparticle solution was injected into the tongue and the left upper limb. Rat B was administered 7.4 MBq of PCSN probes in the tongue and the left upper limb; rat A was injected with a smaller volume (3.7 MBq) in the tongue. The larger injection volume (rat B) revealed an increased uptake in the neck (arrow), with a marked increase in uptake at the site of injection in rat B. A small semiconductor gamma camera clearly displayed the uptake in the neck with an acquisition time of 30 s (A, right). Lymph nodes were dissected from the neck of rat B (**B**, left). Distinct ICG fluorescent lymph nodes in the neck were observed in real time with anatomical resolution (B, right).

**Figure 4:**
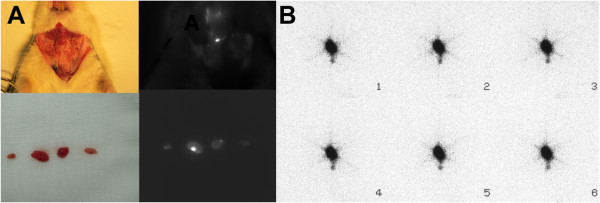
**Lymph nodes from the dissected neck and serial scintigraphy.** Four lymph nodes are shown from the dissected neck of a rat (**A**, upper left). Only one lymph node was observed as fluorescent in the dissected neck (A, upper right). Four lymph nodes were excised (A, lower left). Fine spatial distribution of the fluorescence was observed in one of the four lymph nodes (A, lower right). Serial scintigraphy of a rat after PCSN probe injection. Increased neck accumulation was observed at 10, 20, 30, 40, 50, and 60 min after injection; these time points are numbered 1 to 6, respectively (**B**).

Following the injection of the PCSN probe solution into the rats, two radiologists observed sequential static images on a display at 5-min intervals for 1 h per animal. The scintigraphic images showed a single increased focus of ^99m^Tc activity in the neck with a markedly increased uptake at the injection site (Figures 3 and 4). The fixed increase in uptake observed throughout the study was judged to represent lymph node accumulation. Other migrating or linear uptake was considered to represent movement via lymph channels or flow in the esophagus due to overflow of the PCSN probes at the site of injection (the tongue). A single increased nodal uptake was observed in four of the six injected rats in the final static images. The two remaining animals showed no increase in uptake; however, for one of these animals, this observation was probably due to the injection of an insufficient dose of radioactivity (3.7 MBq of ^99m^Tc as compared with 7.4 MBq in the other rats) (Table 1).

**Table 1:** Summary of the lymphoscintigraphy, contact radiography, and fluorescence imaging findings

**Rat**	**Lymphoscintigraphy**	**Contact radiography**	**NIR fluorescence image**
	**Neck uptake**	**Accumulation**	**Intensity**
Rat A		(−)	(−)
Rat A		(−)	(−)
Rat B	(+)	1, (+)	(+++)
Rat B		2, (+)	(+)
Rat C		1, (−)	(−)
Rat C		2, (−)	(−)
Rat C		3, (−)	(−)
Rat D		1, (−)	(−)
Rat D		2, (slight)	(+)
Rat D	(+)	3, (+)	(++)
Rat D		4, (−)	(−)
Rat E	(+)	1, (slight)	(+)
Rat E		2, (−)	(−)
Rat E		3, (−)	(−)
Rat E		4, (−)	(−)
Rat F		1, (−)	(−)
Rat F	(+)	2, (slight)	(++)
Rat F		3, (−)	(−)
Rat F		4, (−)	(−)

**Figure 5:**
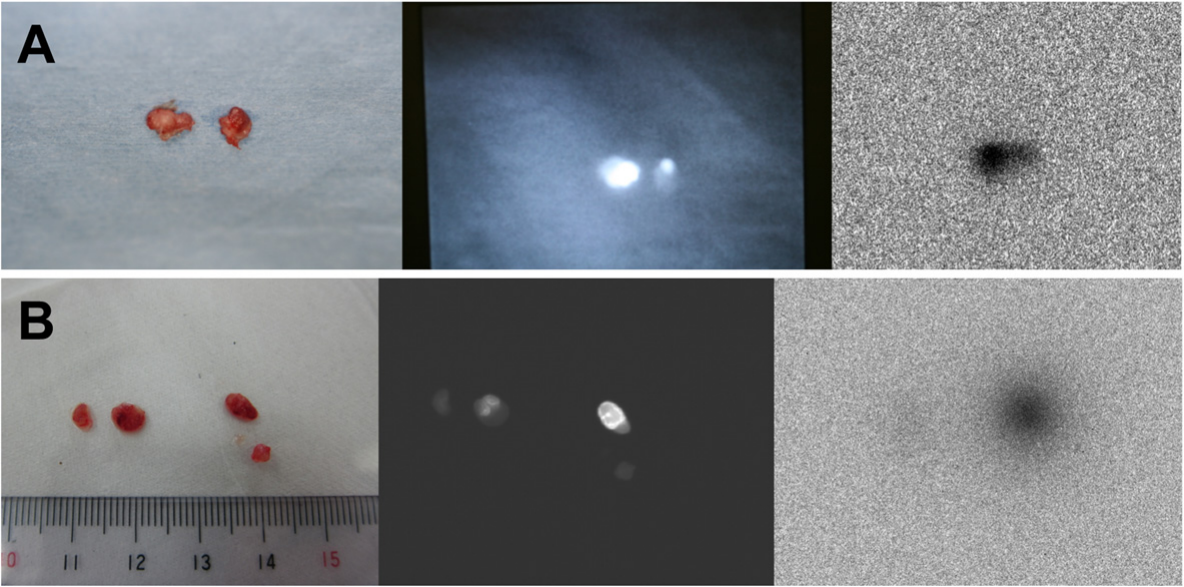
**Excised lymph nodes from rats B and D and contact radiography.** Two excised lymph nodes from rat B are shown (**A**, left). Both lymph nodes were fluorescent (A, center). Contact radiography indicated that the nodes showing increased radioactivity also demonstrated increased levels of fluorescence (A, right). Four excised lymph nodes of rat D are shown (**B**, left). Two of the four lymph nodes were fluorescent. Fine spatial distribution of the fluorescence was observed in the lymph nodes (B, center). Contact radiography indicated a high level of accumulation of ^99m^Tc in the fluorescent lymph nodes (B, right).

Skin incisions were made, and 19 lymph nodes were identified in the six rats (Table 1). The weight (mean ± SD) of these lymph nodes was 12.95 ± 7.76 mg, and the mean of the maximum length of the lymph nodes was 5.1 ± 1.7 mm. Following lymph node excision, contact radiography was performed, which revealed three nodes with marked increases in ^99m^Tc uptake and three nodes with weak uptake (Figure 5).

After skin flaps were created, all the dissected necks were also examined by NIR fluorescence imaging. The PDE system provided clear, real-time fluorescence images of the lymph nodes in the neck with anatomical resolution. NIR fluorescence imaging *ex vivo* was performed following lymph node excision. The intensity of the fluorescence was qualitatively evaluated as weak (+), medium (++), or strong (+++). Six lymph nodes demonstrated weak (+) to strong (+++) fluorescence, and the other lymph nodes showed no fluorescence (−) (Table 1). The precise distribution of the fluorescence was observed inside some of the excised lymph nodes. In one of the rats (rat C), no fluorescent lymph nodes were found in the neck, but faint diffuse fluorescence was observed throughout the cervical soft tissue. In addition, no fluorescent nodes were detected in the neck of rat A due to insufficient injection of labeled probes. For the nodes examined, increased radioactivity was associated with increased fluorescence intensity.

**Figure 6:**
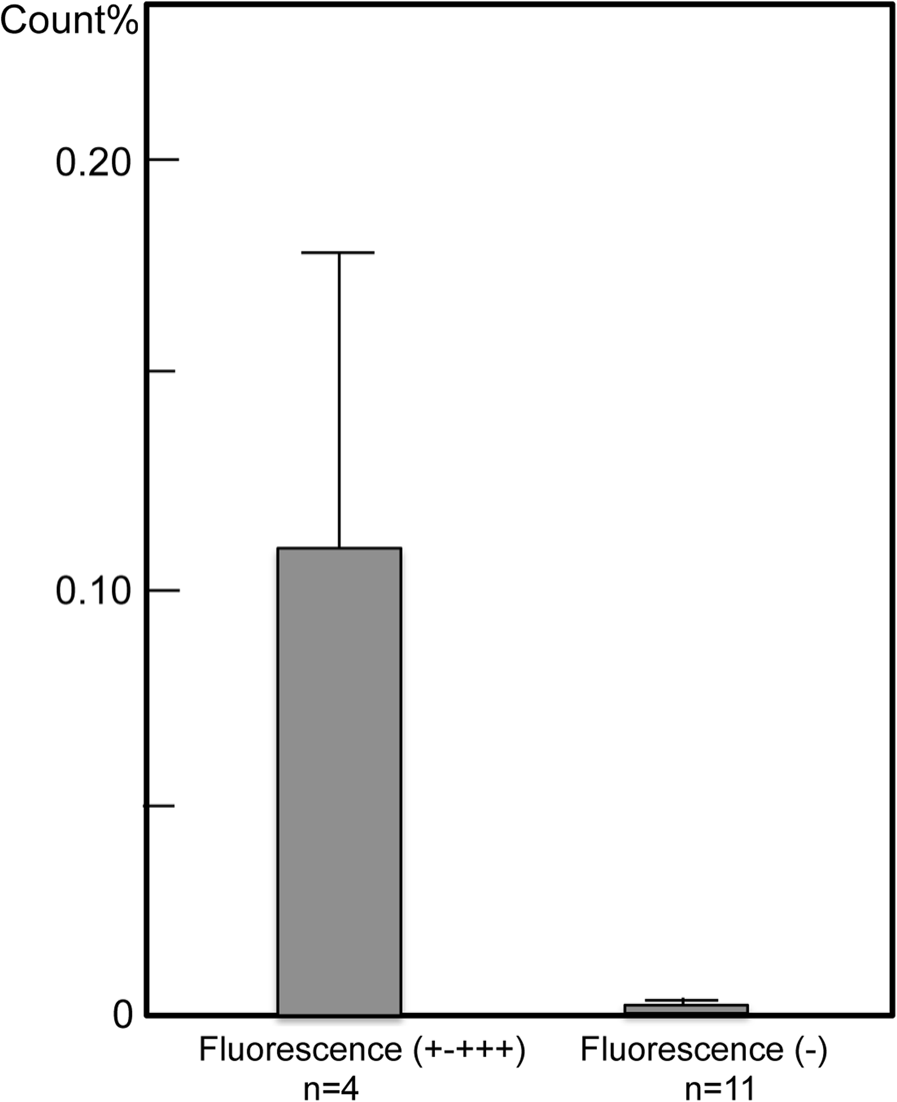
**Fifteen excised lymph nodes were divided into two groups based on their fluorescence intensity.** The radioactivity of the excised lymph nodes relative to the initial injected radioactivity was compared between the high-fluorescence-intensity group (four lymph nodes) and the no-fluorescence-intensity group (11 lymph nodes). The high-fluorescence group also exhibited a high percentage of radioactivity (*p* < 0.05).

The radioactivity of 15 excised lymph nodes from four rats was assayed using a gamma well counter. The lymph nodes of rats A and B were not assayed; there was no specific reason for these animals not being evaluated. The radioactivity of the excised lymph nodes relative to the initially injected radioactivity (7.4 MBq of ^99m^Tc) ranged from 0.231% to 0.000% (Table 2). The 15 excised lymph nodes were divided into two groups according to the intensity of their fluorescence. A comparison of the levels of radioactivity revealed a large difference between the high-fluorescence-intensity group (four lymph nodes; mean, 0.109% ± 0.067%; range, 0.231% to 0.006%) and the no-fluorescence-intensity group (11 lymph nodes; mean, 0.001% ± 0.000%; range, 0.004% to 0.000%). The difference in the means of the fluorescence intensities for the two groups was statistically significant (*p* < 0.05) (Figure 6).

**Table 2:** The percentage of radioactivity in excised lymph nodes relative to the initial injected radioactivity

**Lymph node**	**Radioactivity count**	**%/dose**
Rat C, 1	192.7 ± 6.7	0.001
Rat C, 2	47.3 ± 9.5	0.000
Rat C, 3	1,189.7 ± 31.8	0.004
Rat D, 1	61.3 ± 1.5	0.000
Rat D, 2	6,528.7 ± 135.4	0.020
Rat D, 3	73,784.3 ± 1,043.8	0.231
Rat D, 4	28.3 ± 5.1	0.000
Rat E, 1	13,293.7 ± 451.0	0.012
Rat E, 2	12.3 ± 2.5	0.000
Rat E, 3	10.0 ± 3.0	0.000
Rat E, 4	12.7 ± 0.6	0.000
Rat F, 1	472.7 ± 21.2	0.000
Rat F, 2	6,745.7 ± 392.6	0.006
Rat F, 3	19.7 ± 2.5	0.000
Rat F, 4	10.7 ± 6.7	0.000

Radioactivity was assayed at different times post-injection in rats C, D, E, and F.

**Figure 7:**
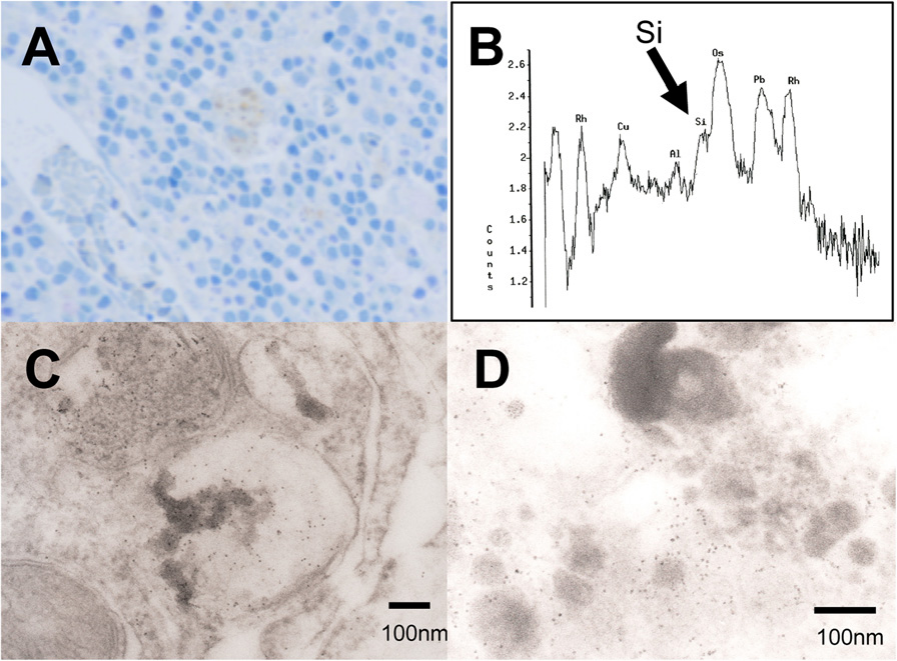
**Toluidine blue staining, TEM of a macrophage, and Energy-dispersive spectroscopy.** Toluidine blue staining shows a blood vessel and macrophages in a lymph node (**A**). TEM of a macrophage (**C**, **D**). High-power images revealed small, black granules in the macrophages. The granules of approximately 10 nm in diameter concentrated in the phagosomes with a neighboring mitochondria that did not contain granules inside (shown at the lower left corner of the panel C). There were also very few granules in the surrounding cellular components (C). Energy-dispersive spectroscopy of TEM. The arrow shows a Si (silicate) peak (**B**).

Regarding the localization of the silica nanoparticles, TEM revealed that small electron-dense granules were localized to the macrophages in seven lymph nodes. The appearance of small spherical granules, 10 to 20 nm in diameter, was restricted to phagosomes of macrophages and was not observed in other lymphatic cells. EDS revealed a Si peak, which suggested that these granules were made of silicon (Figure 7).

## Discussion

There are several advantages to using silica nano-particle-based dual-modality ^99m^Tc/ICG NIR imaging. Preoperative lymphoscintigraphy can reveal deeply situated sentinel lymph nodes, and intraoperative NIR fluorescence imaging provides simultaneous visualization at anatomical resolution in the surgical field following a single injection. These features may facilitate the sentinel lymph node biopsy procedure. Furthermore, it is well known that ambiguous lymphatic drainage results in atypical sentinel lymph node locations [2,18]. Nevertheless, in the cases of unpredictable lymph node drainage basins, surgeons are capable of identifying the sentinel lymph nodes by preoperative lymphoscintigraphy and/or intraoperative surveillance using radiation with greater penetration. In this study, the sentinel lymph nodes were imaged using both radioactivity and NIR fluorescence in the surgical field.

The current study used dual-modality imaging with PCSN probes labeled with ^99m^Tc and NIR fluorescence to evaluate sentinel lymph nodes in the recipient animals. The results demonstrate the potential for these approaches to detect sentinel lymph nodes during clinical biopsy procedures. However, in this animal imaging study, the concentration of the imaging agents substantially affected the image quality. Similarly, it has been reported that very little radioactivity accumulates in the sentinel lymph nodes of human patients [19]. For example, in breast cancer specimens, the administered activities range from 10 to 108 MBq [19]. Although the injected radiopharmaceuticals and the timing of the measurements were different, the mean percentages of the injected doses per lymph node were reported to be 0.9% [20] and 0.60% [21] in patients with breast cancer and 0.34% to 0.92% in studies of cutaneous melanoma [22,23].

In one rabbit study, 5% to 9% (^198^Au-colloid and ^99m^Tc-antimony sulfur colloid) of the injected dose per node was detected [24]. In another rabbit study using 18.5 MBq of ^99m^Tc-phytate, the percentage of the injected dose was 0.21% ± 0.18% (mean ± SD) for lymph nodes in the neck (ranging from 0.01% to 0.62%) [17]. In the current rat study, nodes showing increased uptake contained 0.109% ± 0.067% (mean % per node) of the initial dose of 7.4 MBq. We estimated that, on average, approximately 7.4 KBq of activity accumulated per node. Although this activity is quite small in comparison to that used in clinical practice, the sentinel lymph nodes were accurately visualized using this dose. However, higher doses of ^99m^Tc would have provided much clearer images of the lymph nodes.

We used an NIR imaging video camera system for the *in vivo* imaging; this system has an excitation wavelength of 760 nm and a fluorescence emission wavelength of 820 nm. Before each imaging study, we confirmed that dissolved ICG-sulfo-OSu had bound to the nanoparticles by TLC to verify the absence of free binding spots or flares of fluorescence. The rats were administered a single 0.1-ml submucosal injection of the PCSN probes containing 0.9 μg of ICG-sulfo-OSu. In previous clinical studies, 2.5 mg of ICG was injected for sentinel lymph node mapping using only NIR fluorescence [6,25]. Furthermore, the injection volume was approximately 21- to 210-fold larger than the volume used in our animal experiments. This comparison was calculated using weights of 60 kg for human subjects and 500 g for rats. In addition, the amount injected into the rats was also much less than the amount used in other animal sentinel lymph node studies [26]. However, the comparatively small amount of both the ^99m^Tc and the ICG agents used with the PCSN probes in this study depicted the sentinel lymph nodes successfully.

For sentinel lymph node detection, the size of the radiopharmaceutical is critical for optimal imaging. Multiple sizes have been used for sentinel node detection, including approximately 50- to 100-nm radiolabeled colloid, 50- to 200-nm ^99m^Tc-sulfur colloid, or 4- to 100-nm (95% of the particles <80 nm) ^99m^Tc-nanocolloid of human serum albumin [2]. The PCSN size used in the current study was approximately 30 nm, which is within these previously established ranges. Radiopharmaceuticals have been shown to accumulate, primarily via phagocytosis, in macrophages lining the sinusoid spaces in sentinel lymph nodes [2]. It has been reported that silica-coated fluorescent nanoparticles with a 40-nm diameter were observed in macrophages and dendritic cells of sentinel lymph nodes; phagocytosis of these nanoparticles results in fluorescence enhancement in the lymph nodes [27]. We also detected PCSNs in macrophages, which suggest that retention of these particles may be the result of macrophage phagocytosis.

Optical imaging is typically limited by tissue penetration depth and relatively poor quantification. Fluorescence imaging is limited to tissue depths of 1 to 2 mm in the visible range and as much as 1 to 2 cm in the NIR range [28,29]. Clinically, the detection of the sentinel lymph node using ICG was restricted to 5 mm of tissue depth in oropharyngeal cancer patients [30]. With a wavelength ranging from 750 to 1,000 nm, infrared luminescent light is not directly visible to the human eye. Hemoglobin, water, lipids, and other endogenous chromophores, such as melanin, have their lowest absorption levels within the NIR spectrum, which permits increased photon depth penetration into the tissues [31]. Furthermore, in the NIR spectrum, the autofluorescence emitted from endogenous fluorophores, such as NADH and porphyrins, is extremely low compared with UV and visual light wavelengths, which provides an improved signal-to-background ratio. By contrast, ICG emits infrared fluorescence.

Successful combined imaging using ^99m^Tc and ICG fluorescence for sentinel biopsy of the lymph node has been reported recently [10-12]. Incubation of ^99m^Tc-nanocolloid and ICG provided a favorable agent retention time, with the fluorescence lasting up to 19 h post-injection, for the detection of sentinel lymph nodes in the necks of patients with squamous cell carcinoma of the oral cavity [32]. In addition, another new NIR imaging approach to visualize sentinel lymph nodes has been demonstrated in animal studies using nano-carriers, such as quantum dots [31,33-35] and dendrimers [36-38].

Silica nanoparticles have also been used for pre-clinical *in vivo* fluorescence imaging [39]; these particles are also attractive for approaches aiming to integrate multiple properties for multimodality biomedical imaging [40]. Furthermore, functionalized organically modified silica nanoparticles can serve as an ideal nano-platform for the assembly of multimodal nanoparticles for targeted diagnostics and therapeutics [41]. Benezra et al. used dye-encapsulating silica particles that were surface-functionalized with cyclic arginine-glycine-aspartic acid peptide ligands and radioiodine, which exhibited high-affinity/avidity binding to α_v_β_3_ integrin in mouse melanoma [42].

The Aerosil 200 used in this study is an amorphous, fumed hydrophilic silica. Of the reported nanoparticles used for imaging, amorphous silica nanoparticles are feasible materials for *in vivo* imaging due to their biocompatible nature compared to other agents, including quantum dots, which contain cadmium telluride crystals. However, it has been reported that amorphous silica and crystalline silica are phagocytosed and are therefore toxic to mouse alveolar macrophage cells. In addition, the inhalation of crystalline silica has been shown to cause silicosis [43]. Nevertheless, the cytotoxicity induced by nano-scale silica particles is complex and depends on the particle size, the surface functionalization, and the cell line used for the study of toxicity [44].

The PCSNs used in this study were functionalized using PAMAM, which is a type of dendrimer that is characterized as a branched synthetic molecule composed of spherical nano-structures with a high degree of molecular uniformity. Importantly, PAMAM possesses the potential to contribute to *in vivo* imaging and drug delivery vehicles as the dendrimer can be used to generate highly functionalized terminal surfaces [45]. We used the terminal NH_2_ PAMAM for covalent bonding between the PCSN surfaces and ICG with simultaneous ^99m^Tc chelation. Thus, our PCSN probe may help to convey drugs, antibodies, and ligand molecules by acting as a targeted carrier. It is known that the flexibility of dendrimers, along with their size, charge, and nanoparticle hydrophilicity, influences their imaging potential by changing their biodistribution and pharmacokinetics [46]. It is assumed that because our PCSNs comprise the silica core and surface dendrimers, the PCSN probes may exhibit stable biological characteristics due to the lower flexibility of the dendrimers in contrast to probes solely composed of PAMAM dendrimers. However, how this difference would affect *in vivo* imaging is not well known. Further elucidation will require more study.

## Conclusion

In this animal study, surface-functionalized silica nanoparticles clearly depicted sentinel lymph nodes in real time with the use of dual-modal imaging using a low concentration of ^99m^Tc and ICG NIR fluorescence. This approach may facilitate sentinel lymph node biopsy procedures, and it represents a novel method for targeting metastatic cells for imaging and therapy.

## Competing interests

The authors declare that they have no competing interests.

## Authors’ contributions

MaT participated in the design of the study, performed the statistical analyses, and drafted the manuscript. MaT, KH, and MiT participated in the conduct of the study. IS performed the TEM studies. NT prepared the PAMAM-grafted silica nanoparticles and executed the zeta potential measurements and the SEM studies. MaT and KH participated in the study coordination, image analysis, and data analysis. All authors read and approved the final manuscript.
